# Changes in biochemical composition of Ethiopian *Coffee arabica* with growing region and traditional roasting

**DOI:** 10.3389/fnut.2024.1390515

**Published:** 2024-05-29

**Authors:** Dhaba Mengesha, Negussie Retta, Henock Woldemichael Woldemariam, Paulos Getachew

**Affiliations:** ^1^Center for Food Science and Nutrition, College of Natural and Computational Sciences, Addis Ababa University, Addis Ababa, Ethiopia; ^2^Ethiopian Institute of Agricultural Research (EIAR), Addis Ababa, Ethiopia; ^3^Department of Food Engineering, College of Biological and Chemical Engineering, Addis Ababa Science and Technology University, Addis Ababa, Ethiopia

**Keywords:** Ethiopian coffee varieties, roasting, biochemical composition, antioxidant capacity, *Coffee arabica*

## Abstract

Updating the biochemical composition of coffee beans across the years is necessary. This is important to understand the vulnerability of coffee toward climate adaptation longitudinally. Accordingly, in this study the influence of growing area and traditional roasting on the biochemical composition of five common Ethiopian Arabica coffee beans collected in the harvest year of 2021/22 were investigated. With an average of 11.34 g/100 g, the Hararge and Jimma coffee beans had the highest crude fat content (*p* < 0.05). The crude protein content of the five varieties was in the range of 13–15 g/100 g, with respective highest and lowest contents in the (Hararge and Nekemte) and (Sidama and Yirgachefe) coffee beans (*p* < 0.05). The total phenolic content (TPC) of the coffee beans was in the order of Jimma (46.52) > Nekemte (44.55) > Sidama (44.31) > Hararge (39.02) > Yirgachefe (34.25) mg GAE/100 g. The 50% inhibitory concentration (IC50) of ascorbic acid, coffee bean extract from Jimma and Hararge against 2,2-diphenyl-1-picrylhydrazyl (DPPH) free radical was 19.86, 20.22 and 20.02 μg/mL, respectively. The respective highest and lowest caffeine concentration was obtained in the Yirgachefe (10.38) and Hararge (7.55 g/100 g) coffee beans (*p* < 0.05). The Jimma, Sidama, and Nekemte coffee varieties had the highest chlorogenic acid content of 45 g/100 g (*p* > 0.05); whereas the lowest content was in Hararge coffee (36.78 g/100 g). While the caffeine concentration did not show significant (*p* > 0.05) difference, with all the coffee beans the roasting has reduced significantly the TPC, trigonelline and mainly the chlorogenic acid (*p* < 0.05). These data can update the existing facts on biochemical diversity of coffee beans in the country which can be used for evidence based innovations of climate adaptation in predicting the quality of coffee. Further recommendation of optimizing the traditional coffee processing method is supported from this study.

## Introduction

Coffee is one of the most widely sold and consumed drinks worldwide (1, 2). It is valued for its flavor, pleasant aroma, invigorating impact, and several health benefits (3, 4). The world’s coffee production amounted to 70% is derived from *Coffea arabica*. The remaining 30% is produced by *Coffea canephora* and *Coffea liberica* (5, 6). *Coffea arabica* originated in Ethiopia and there is significant genetic diversity in the country. Ethiopia is the highest producer of coffee in Africa and the fifth major exporter in the world next to Brazil, Vietnam, Colombia, and Indonesia, contributing to 4.2% of the total world coffee production (7). According to Tefera et al. (8) and Worku (9), the coffee industry in Ethiopia leads the agriculture sector in terms of its contribution to the national economy overall and to exports in particular. Coffee’s quality, which is determined by its biochemical makeup, is one of the key factors that affect its market price and customer preference. For example, there is a considerable correlation between high amounts of caffeine and trigonelline coffee beans and high-quality cups (10).

Because Ethiopia has diverse agro-ecologies, its farmers are able to produce high-quality coffee with a range of biochemical compositions (11, 12). Thus, in each coffee region, different coffee varieties that are thought to have distinct quality attributes are recognized in the local and/or international markets; these include the coffees of Harar in the east, Sidamo, Yirgacheffe, Guji, and Kochere in the south, Jimma, Limmu, Kaffa, Bebeka, and Tepi in the southwest, Lekemt (Wellega) coffee in the west, and Wenbera and Zegie coffees in the northwest (13). Geography is one of the environmental factors affecting coffee quality as areas suitable for agriculture are shifting with climate change. Hence, cultivating coffee in different geographic areas is associated with variation of key secondary metabolites driving its quality. However, research gaps were found regarding the effects of climate change on the directionality of coffee quality and how this varies with location, elevation, and management conditions (14). For this purpose, updating the existing biochemical composition and quality attributes of coffee beans across the years is necessary. Besides, despite several studies reported on the coffee quality performance in different regions of Ethiopia (12, 15, 16), there is no comprehensive research on the biochemical composition (macronutrients, bioactive compounds) and antioxidant activity of Ethiopian coffee across coffee-growing regions as to our knowledge.

The majority of chemicals associated with coffee’s aroma are formed when the green bean is partially destroyed during roasting via Maillard reaction. Hence, the roasting of coffee alters the biochemical composition, such as free amino acids, sugar, chlorogenic acids and trigonelline content (17–19) as well as sensory quality (20–22). High levels of caffeoylquinic acids, feruloylquinic acids and their oxidation products are, however, associated with poor cup quality and off-flavor (13). Therefore, the study on the influence of coffee roasting on the biochemical composition is necessary. However, there are limited reports on the influence of Ethiopian traditional roasting on the biochemical compositions of the mentioned coffee varieties.

Therefore, this study assessed the effect of coffee origins from the top five major coffee producing areas of Ethiopia (Jimma/Limu, Sidama, Hararge, Nekemte, and Yirgacheffe) and traditional roasting on the its biochemical composition and quality attributes.

## Materials and methods

### Climate characteristic of the study areas (2021/22)

Hararge zone: Hararge is in the eastern highlands of Ethiopia with an elevation ranging between 974 and 3,264 m mean above sea level (m.a.s.l) (average 2047 m). The mean annual rainfall of the area is 820.01 mm. The mean annual temperature is around 15–20°C. The soil type in this study area is sandy, clay loam with the sand dominating the proportion (23).

Sidama zone: this study area is located in the Sidama regional state which is located in the southern part of the country. Altitude ranges from 560 to 2,300 m m.a.s.l (average 1,694 m) with the average annual temperature of 27.2°C. The average annual rainfall ranges between 700 and 1,200 mm. The dominant soil types in this study area are clay, silt and sand, respectively. The rainfall pattern in the area is bimodal, which occurs in the summer and spring seasons (24).

Yirgachefe zone: located in the Gedeo Zone of the Southern Nations, Nationalities and Peoples’ Region (SNNPR) of Ethiopia. This town has an elevation between 200 to 1,919 m m.a.s.l (average 1,570 m). The mean annual temperature of the zone range from 18 to 25°C with annual rainfall varying from 150 to 1,000 mm per year. Nitisols are the dominant soil type in this area (25).

Jimma zone: Jimma is located in the Western part of the country with altitude range between 1,200 to 3,020 m m.a.s.l (average 1,657 m), where the rainfall amounts to 1,500 mm per year. Jimma is very warm year-round, with the daily maximum temperature between 24 and 27°C. Nitisols are the dominant soil types in the zone (26, 27).

East Wollega zone: the altitude of the study area ranges from 1,300–3,140 m m.a.s.l (average 2,123 m), and the district has various topographic features. The area has average annual temperature of 21.5°C with annual rainfall between 291.8 to 1325.6 mm. The dominant soil type in the area is nitisol (28, 29).

### Sample collection

In this study, five *Coffee arabica* varieties (Jimma/Limu, Sidama, Hararge, Nekemte, and Yirgacheffe) were selected because they are top highly produced and consumed varieties in Ethiopia. Furthermore, these varieties grow in different agro-ecological areas, which is the core to address the objective of the study to evaluate its influence on the biochemical composition of the beans. From each variety a composite sample was collected from the major producing sites of the agro-ecological regions. The sampling was replicated in time and space three times in the harvest year of 2021/22. The samples were collected unwashed or dry-processed for wholesome coffee quality. The raw coffee bean varieties were physically examined for defects, odor quality, size, and shape, then packed and stored in polyethylene bag.

### Sample preparation

The coffee samples were subdivided into two for green and roasted bean analysis. About 100 g coffee beans were ground to a fine particle size (<0.5 mm) using a coffee grinder (Probat BRZ6, Germany) for biochemical composition analysis. The other half, 100 g of green coffee beans were roasted at 190°C on Ethiopian traditional coffee roaster (earthenware) to a medium level for 16 min. Then, the roasted coffee was grounded (~0.75 mm) using an electrical coffee grinder (Mahlkoing, Germany) after air-cooling.

### Proximate analysis

The moisture, crude protein, crude fat, and total ash contents of the coffee samples were analyzed according to the methods of the Association of Official Analytical Chemists (30). Moisture content was determined by oven drying at 105°C to constant weight (protocol no: AOAC. 925.10). The crude protein content (*N* × 6.25) was determined by the Kjeldahl method based on determination of nitrogen content (AOAC. 981:10) (INOK1160 Automatic Kjeldahl Protein/Nitrogen Analyzer). The crude fat content was determined using the semi-automatic Soxtec system (AOAC 991:36; Barnstead Electro-thermal, Staffordshire, United Kingdom). Total ash was determined gravimetrically in a heated muffle furnace (Muffle furnace size 2, England) at 550°C. Total carbohydrate content was calculated by difference as follows:


(1) $$\mathrm{Total}\;\mathrm{carbohydrate}\;\left(\%\right)=\left(100-\left(\mathrm{moisture}+\mathrm{ash}+\mathrm{protein}+\mathrm{fat}\right)\%\right)$$

The gross energy was calculated from protein, fat, and carbohydrate values, as follows:


(2) $$\begin{array}{l} \mathrm{Gross}\;\mathrm{energy}\;\left(\mathrm{kcal}/100\;g\right)=\\ \left(4*\mathrm{protein}\;\left(\%\right)+4*\mathrm{carbohydrate}\;\left(\%\right)+9*\mathrm{fat}\;\left(\%\right)\right) \end{array}$$

### Sample extraction for bioactive compounds

Total phenolic, flavonoid content, antioxidant activity and bioactive compounds in the green and roasted coffee were extracted using the methodology by Amigo-Benavent et al. (31) with some modifications. Briefly, 3 g of each powder sample was mixed with 20 mL of 80% methanol and placed in a shaking incubator (Lucadema, Brazil) at 40°C and 120 rpm for 30 min. Then, the content was centrifuged for 15 min at 114 g in an Excelsa Baby II centrifuge (Fanem, model 206-R). The supernatant was used to determine the phenolic compounds. Each extraction was carried out in triplicate.

### Determination of total polyphenol content

The TPC was estimated by the Folin–Ciocalteu method (32). Gallic acid was used to make the standard curve for quantification of TPC. Gallic acid standard solutions: 2, 4, 6, 8, 10, and 12 mg of gallic acid were weighed and one milliliter of ethanol was added. This was followed by adding 3 mL of distilled water. Then, 0.1 mL of Folin–Ciocalteu reagent was mixed. After 3 min, 0.3 mL of 2% sodium carbonate solution was followed and the volumetric flasks were adjusted to the mark with ethanol. The whole content was kept at 25°C for 2 h. Then, the wavelength of the spectrophotometer was adjusted to 765 nm. At first, the device was zeroed with ethanol, and then the absorbance of each of the solutions was read three times and the average was recorded. Then, standard curve was developed and the line equation was used for the quantification.

For the coffee samples, 10 mg of the green bean extract was weighed, placed in a test tube, and 2 mL of dimethyl sulfoxide (DMSO) solution was added to completely dissolve the extract. For each extract, three 5 mL volumetric flasks were used for triplicate measurements. Each of the volumetric flasks contained 3 mL of distilled water and 0.20 mL of each extract. Then, 100 μL of Folin–Ciocalteu reagent was added and after 3 min, 0.3 mL sodium carbonate (2%) followed. Then, the volumetric flasks were adjusted to the mark with distilled water. A 5 mL control volumetric flask containing 0.20 mL of DMSO instead of the extract was used as control. The whole content was homogenized and placed at 25°C for 2 h. Then, the absorbance of each solution was read three times using a UV–Vis spectrophotometer (Model UV-2100, United States) at 765 nm. The TPC was expressed as mg of GAE/100 g of extract (Eq. 3).


(3) $$TPC=\frac{C*V}{M}\times D$$

where;

C = gallic acid equivalent concentration obtained from the calibration curve (g/mL).

V = volume of the extract’s stock solution (mL).

M = dry weight of the extract in the stock solution (g).

D = dilution factor.

### Total flavonoids content

The TFC was determined by applying the aluminum chloride colorimetric method according to Magalhães et al. (33). Flavonoid concentration was deduced from the range of the calibration curve established using quercetin. The total flavonoid contents of the green coffee beans were determined using a UV–Vis spectrometer (Model UV-2100, Made in the United States) (34). Briefly, a 20 μL aliquot of the coffee extract (prepared as mentioned above) was mixed with 20 μL of 5% NaNO_2_ solution and incubated for 5 min. Subsequently, a 40 μL aliquot of 10% aqueous solution of AlCl_3_, 75 μL of 1 M aqueous solution of NaOH, and 100 μL of distilled water were added, and the absorbance of the resulting mixture was recorded at 415 nm. Distilled water (20 μL) treated similarly as the samples was used as a blank. Quercetin was used as the reference standard to express the total flavonoid content of the coffee samples. A stock solution of quercetin (500 mg/L) was prepared by dissolving 50 mg in 100 mL of 5% aqueous methanol. The calibration standards (25–250 mg/L) were treated with the same reagents and incubated under identical conditions as the samples to develop the calibration curve. The total flavonoid contents of the coffee samples were expressed as mg quercetin equivalents (QE) per 100 g of dry mass. The analysis was done in triplicate.

### Anti-oxidant assay using DPPH scavenging capacity

The ability of the coffee extracts to scavenge the free radical 2,2-diphenyl-1-picrylhydrazyl (DPPH) was measured according to the method by Fukumoto and Mazza (35). One mL of DPPH solution and 1 mL of methanol were mixed and stored at 25°C for 30 min. Then, using a UV–Vis spectrophotometer (Model UV-2100, United States), the absorption of the solution was measured at 517 nm. Ascorbic acid (standard), 25 mg was weighed and completely dissolved in 25 mL of methanol in a 25 mL volumetric flask (1 mg/mL). Then, concentrations of 0.02, 0.04, 0.08, 0.12, 0.16, and 0.20 mg/mL of the solution from the stock solution were prepared. To prepare the sample solution, 25 mg of each of the methanol extracts from the coffee samples was weighed, poured into a 25 mL volumetric flask, and mixed with methanol. Then, the whole content was well homogenized. The stock solution was prepared at a concentration of 1 mg/mL. Six solutions with concentrations of 0.02, 0.04, 0.08, 0.12, 0.16, and 0.20 mg/mL were prepared. One milliliter of each of the above solutions was mixed with 1 mL of the DPPH solution and kept at 25°C for 30 min. Then, the absorption of the solution at 517 nm wavelength was read using UV/Vis spectrophotometer, starting with the control solution and then reading the samples in order of increasing concentration. The analysis was replicated three times, and after averaging the data, the 50% inhibitory concentration of the extracts to scavenge the radical (IC50) was determined. The percentage of inhibition was calculated using the following equation:


(4) $$\begin{array}{l} \%\mathrm{Inhibition}=[\left(\mathrm{Abs}.\mathrm{of}\;\mathrm{control}-\mathrm{Abs}.\mathrm{of}\;\mathrm{test}\;\mathrm{sample}\right)\\ ÷\mathrm{Abs}.\mathrm{of}\;\mathrm{control}]*100\% \end{array}$$

where:

Abs = absorbance.

### Bioactive compounds analysis

#### Extraction of caffeine, trigonelline, and chlorogenic acids

Caffeine (CAF), chlorogenic acid (CGA), and trigonelline (TRG) extractions were done following the method by Heo et al. (36) with some modifications. About 0.5 g finely ground coffee powder was accurately weighed into a 50 mL Erlenmeyer flask. Heated (95°C) distilled water (50 mL) was added and stirred for 20 min. Then, the extract was filtered using Whatman No. 4 filter paper and subsequently through a 0.22 μm pore size and a 10 μL specimen was injected into the high performance liquid chromatography (HPLC) (Agilent 1,260 Infinity, Germany).

#### Determination of trigonelline, caffeine, and chlorogenic acid

Simultaneous determination of TRG, CGA, and CAF was done using the HPLC system consisting of a Discovery C18 column with an gradient flow rate of 0.7 mL/min and a methodology adapted from Heo et al. (36) with some modifications. The column used was a 4.6 × 250 mm with 5 μm particle size (Waters, Taunton, United States). Elute solvents containing 5% acetic acid (A) and acetonitrile (B) were as follows: 0–4 min: 4% B; 4–8 min: 10% B; 8–12 min: 90% B; 12–15 min: 0% B; and 15–17 min: 4% B, at a flow rate of 0.7 mL/min. A 3 min post-run was used. TRG and CAF were detected at 272 nm, while CGA was detected at 320 nm. Triplicate measurements were used for quantification of these compounds. TRG and CAF were evaluated at 5, 10, 20, 40, 50, 100, and 150 μg/mL. Whereas, CGA was evaluated over the range of 5, 10, 20, 100, 150, and 200 μg/mL. CAF, TRG, and CGA were identified by comparing the retention times of the CAF standard (99%) (Fischer Scientific), TRG standard (Sigma Aldrich), and CGA standard (Acros organics) and their concentrations calculated from peak areas using calibration equations. The limits of detection (LOD) and quantification (LOQ) for caffeine, trigonelline and chlorogenic acid were (0.90, 1.70), (1.02, 2.30), and (1.50, 3.00) μg/g, respectively.

### Statistical data analysis

The results were expressed as the mean and standard deviation of three different determinations. Data analysis was performed by using the statistical software package SPSS 23 (IBM Corp, United States). Analysis of variance (ANOVA) was used to test significant differences at *p* < 0.05 between mean values of biochemical composition of green beans among the growing regions and upon traditional roasting.

## Results and discussion

### Macronutrient composition

One of the most important factors supporting producers like Ethiopia succeed in the global coffee market is coffee quality. More crucially, as is commonly seen in the present global market, coffee quality dictates its relative price. However, climate-driven changes are impacting crops in multiple ways including changes in the geographic ranges suitable for cultivation and crop quality (14). Therefore, continuous evaluation of the biochemical makeup of coffee varieties in different geographical locations is important to model the longitudinal impact of climate change on coffee quality compositions. These quality parameters include physical, organoleptic, and biochemical components (i.e., macro- and micronutrients, and secondary metabolites) (15).

In this study, the macronutrient composition of the different agro-ecological origin Ethiopian *C. arabica* varieties is presented in Table 1. The green *C. arabica* from the Hararge and Jimma origins had the highest crude fat content (*p* < 0.05), averaging 11.34 g/100 g. Likewise, Ahmed et al. (14) reported higher lipid content in coffee beans with higher altitude areas like Hararge. The average lipid content of green coffee beans was reported by Risticevic et al. (37) to be between 13 and 17 g/100 g, which are higher than the values found in the current study. Coffee from the Sidama origin had the highest crude fiber content (30 g/100 g; *p* < 0.05), whereas coffee from the Hararge origin had the lowest value (25 g/100 g). At 4 g/100 g (*p* < 0.05), the Nekemte coffee beans had the highest total ash level (Table 1). Numerous studies showed that growing coffee in various regions led to variations in a number of primary metabolites, such as lipids, sugars, amino acids, and minerals. For example, when *Coffea Arabica* is transplanted outside of its place of origin, Ethiopia, lipid content decreased (14). Thus, geographical origin, fertilizer usage, and genetics are thought to affect coffee quality and biochemical composition (13, 38, 39).

**Table 1:** Macronutrient composition of Ethiopian green *Coffee arabica* varieties grown in different agro-ecological areas.

Coffee origin	Moisture (g/100 g)	Crude fat (g/100 g)	Total ash (g/100 g)	Crude protein (g/100 g)	Crude fiber (g/100 g)	Total carbohydrate (g/100 g)	Gross energy (kcal/100 g)
Jimma	7.00 ± 0.39^ab^	11.24 ± 0.02^a^	3.00 ± 0.39^b^	14.00 ± 0.00^b^	26.00 ± 0.19^cd^	64.20 ± 0.19^c^	413.96 ± 4.04^b^
Sidama	7.00 ± 0.19^ab^	10.23 ± 0.02^e^	3.00 ± 0.00^b^	13.00 ± 0.00^c^	30.00 ± 0.00^a^	66.77 ± 0.39^b^	411.15 ± 6.29^c^
Yirgachefe	6.00 ± 0.59^ab^	10.85 ± 0.07^c^	3.00 ± 0.19^b^	13.00 ± 0.19^c^	29.00 ± 1.18^b^	67.15 ± 0.19^a^	418.25 ± 4.59^a^
Nekemte	6.00 ± 0.19^ab^	10.67 ± 0.03^d^	4.00 ± 0.78^a^	15.00 ± 0.19^a^	26.00 ± 0.19^cd^	64.33 ± 0.19^c^	413.35 ± 4.76^b^
Hararge	8.00 ± 0.71^a^	11.44 ± 0.09^a^	3.00 ± 0.39^b^	15.00 ± 0.00^a^	25.00 ± 0.19^d^	62.56 ± 0.39^d^	413.20 ± 6.76^b^

Data are expressed as mean ± SD, (*n* = 3). Mean values within a column with different superscript letters indicate a significant difference at *p* < 0.05 in mean comparison using Duncan multiple range test.

Proteins are known flavor precursors in coffee being denatured and fragmented during roasting (40). Hence, evaluating the protein content of coffee beans is important across different agro-ecological origins. Accordingly, the crude protein content of the five Ethiopian *C. arabica* varieties was in the range of 13–15 g/100 g (Table 1). These values are within the range reported by Oliveira et al. (41), 11.0–16.5 g/100 g. Comparably, the crude protein level (14.9–17.0 g/100 g) matched the values reported in commercial coffee beans grown across Brazil (42). In this study, the highest and lowest crude protein content was obtained in the Hararge and Nekemte (15 g/100 g) and Sidama and Yirgachefe (13 g/100 g) coffee beans (*p* < 0.05). Degefa et al. (7) reported the protein content of 104 coffee bean samples from Ethiopia for different coffee genotypes ranged from 6.93–10.14 g/100 g. The highest carbohydrate content (67.15 g/100 g) was obtained in the Yirgachefe coffee. The altitude at which coffee is cultivated is the most significant environmental component that is frequently associated with coffee quality. The lowest daily temperatures at higher altitudes produce *Arabica* coffee of the highest quality because they cause the beans to ripen more slowly and provide bean filling more time. Thus, a longer maturation period increases the plant’s likelihood of obtaining enough minerals and nutrients from the soil. More buildup of biochemical compositions will result from this (43). This might be the case of the Hararge coffee’s better composition of the majority of macronutrients (Table 1).

## Total phenolic, flavonoid, chlorogenic acid, trigonelline, and caffeine content

Coffee is a rich source of bioactive compounds especially polyphenols. These compounds contribute to the total polyphenol intake in diet and are beneficial to consumer health (44). The total phenolic content (TPC) of the coffee beans was in the order of Jimma (46.52) > Nekemte (44.55) > Sidama (44.31) > Hararge (39.02) > Yirgacheffe (34.25) mg GAE/100 g (Table 2). Likewise, there was a variation in the total flavonoid content (TFC) in order of Nekemte (50.10) > Jimma (45.10) > Yirgacheffe (37.80) > Sidama (37.30) > Hararge (31.50) mg QE/100 g. The Jimma and Nekemte coffee varieties had the highest TPC and TFC, respectively, (*p* < 0.05) (Table 2). Likewise, Masek’s et al. (45) investigation on five distinct Ethiopian coffee brands revealed a noteworthy total phenolic content and antioxidant activities, suggesting potential applications for Ethiopian coffee in the prevention and treatment of a number of degenerative disorders.

**Table 2:** Total phenolic, flavonoid, chlorogenic acid, trigonelline, and caffeine content of Ethiopian green *Coffee arabica* varieties grown in different agro-ecological areas.

Bioactive compounds	Jimma coffee	Sidama coffee	Yirgachefe coffee	Nekemte coffee	Hararge coffee
TPC (mg GAE/100 g)	46.52 ± 0.82^a^	44.31 ± 4.68^ab^	34.25 ± 3.83^c^	44.55 ± 3.28^ab^	39.02 ± 2.93^bc^
TFC (mg QE/100 g)	45.10 ± 0.36^ab^	37.30 ± 0.75^bc^	37.80 ± 0.55^bc^	50.10 ± 0.23^a^	31.50 ± 0.57^c^
Caffeine (g/100 g)	8.85 ± 0.00^b^	8.23 ± 0.00^c^	10.38 ± 0.00^a^	10.18 ± 0.00^a^	7.55 ± 0.00^e^
Chlorogenic acid(g/100 g)	45.95 ± 0.01^a^	45.29 ± 0.01^a^	39.54 ± 0.01^b^	46.39 ± 0.01^a^	36.78 ± 0.02^c^
Trigonelline (mg/100 g)	12.88 ± 0.01^ab^	13.56 ± 0.02^a^	13.46 ± 0.01^a^	11.78 ± 0.00^b^	11.65 ± 0.00^b^

Data are expressed as mean ± standard deviation (*n* = 3) on dry basis. Mean values within a row with different superscripts indicate significant difference at *p* < 0.05 using one-way-analysis of variance (ANOVA), in mean comparisons using Duncan’s multiple range test. TPC, total phenolic content; TFC, total flavonoid content; GAE, gallic acid equivalent; QE, quercetin equivalent.

The cup quality of coffee is influenced by a number of phytochemicals, including sugar levels, oil, methylxanthines, and chlorogenic acid (46). However, according to Sualeh et al. (15), the most often used standards for assessing the quality of coffee are caffeine, trigonelline, and chlorogenic acids. Therefore, in this study, the influence of agro-ecology on these biochemical makeups of coffee was evaluated. The caffeine content of the five Ethiopian *C. arabica* varieties was in the range of 7.55–10.38 g/100 g. The respective highest and lowest caffeine concentration was obtained in the Yirgachefe and Hararge coffee beans (*p* < 0.05). Except between Nekemte and Yirgachefe varieties, between all there was a significant difference in caffeine content (*p* < 0.05). Coffee’s distinctive bitterness is attributed to caffeine, an alkaloid and nitrogenous secondary metabolite (47). Generally, the higher the caffeine content the bitter is its taste and thus the lower is its cup quality (48). Similarly, Sualeh et al. (15) reported a significant variation in caffeine content of coffee beans collected from nine districts of Southwest Ethiopia (i.e.0.85–1.73 g/100 g). This value is lower than in the present study. Also the caffeine content in this study is higher than the report by Farah (49) (i.e., 0.9–2.5 g/100 g) in different coffee varieties. Likewise, Worku et al. (13) reported that caffeine content was the most varied variable among the localities across the five coffee regions in Ethiopia. In less popular coffee growing region of Ethiopia, Amhara region (northern Ethiopia), Wale et al. (50) reported the caffeine content ranged from 0.78 to 1.55 g/100 g in green coffee beans grown in different zones of the region. These values are lower than in the present study.

The main phenolic components of green coffee are chlorogenic acids. These are quinic acid and trans-hydroxycinnamic acid esters (46). These chemical groups are recognized to be in charge of giving coffee beverages their color, flavor, bitterness, and astringency (51). Chlorogenic acids make up about 6–12% of the dry weight of green coffee beans (52). In this study, the Jimma, Sidama, and Nekemte coffee varieties had the highest chlorogenic acid (*p* > 0.05) 45 g/100 g compared with the other two (*p* > 0.05). The Hararge variety had the lowest chlorogenic acid, 36.78 g/100 g (Table 2). The chlorogenic acid concentration of a given coffee variety has inversely correlated with its beverage quality where higher content was observed in coffee varieties that had lower beverage quality (48). The chlorogenic acid content ranged between (2.80–5.42 g/100 g) in the green coffee beans collected from Southwest Ethiopia nine districts (15). In the green coffee beans collected from the northern part of Ethiopia, Wale et al. (50) reported chlorogenic acids content ranged from 3.29–7.73 g/100 g. Farah (49) reported chlorogenic acid contents of different coffee varieties within the range of (4.1–11.3 g/100 g). All these prior studies reported a lower content of chlorogenic acid than the present study. Chlorogenic acids have strong antiviral, antidiabetic, antioxidant, and neuroprotective activity (53).

Similarly, the Jimma, Sidama, and Yirgachefe coffee varieties had the highest trigonelline contents of 12.88, 13.56, and 13.46 mg/100 g, respectively (*p* > 0.05). The lowest concentration was reported in the Nekemte and Hararge varieties (11.78 and 11.65 mg/100 g respectively, *p* > 0.05) (Table 2). Sualeh et al. (15) reported a significant variation in trigonelline content of coffee beans collected from nine districts of Southwest Ethiopia (i.e., 0.80 to 1.08 g/100 g). Wale et al. (50) reported trigonelline content of 0.53–1.27 g/100 g in green coffee beans collected from the northern part of the country. These prior studies reported lower values of trigonelline than the present study indicating its dependence on agro-ecological variation. According to Mehari et al. (46), trigonelline, also known as N-methylpyridinium-3-carboxylate, is the second most common alkaloid in coffee, behind caffeine. Although trigonelline can also be found in barley, corn, onions, peas, soybeans, and tomatoes, coffee is the main source of this compound (54).

Overall, the variations observed among the coffee regions in their TPC, TFC, caffeine, chlorogenic acid, and trigonelline content can be linked to the variation in growing environment conditions (i.e., altitude, soil type, rainfall and other agricultural practices) (15). For example, the southwestern, western and northwestern regions have a higher total rainfall and a longer rainy period than Hararge and southern regions (13). This highly influences the TPC (55). These environmental-driven changes in crop quality may be offset by climate adaptation strategies and other management conditions. However, fewer studies have focused on effects on crop quality (14). For instance, as such geographic variations resulted in shifts in important odor-active markers of coffee including the volatiles 2,3-butanedione, 2,3-pentanedione, 2-methylbutanal, and 2,3-dimethylpyrazine (14). Therefore, across the years evaluation of the biochemical composition of coffee beans is necessary for evidence based innovations of climate adaptation in predicting the quality of coffee. This study is approached in such a way to contribute to the database, besides evaluating the influence of geographical origins on biochemical composition.

### DPPH radical scavenging activity

With concentration the DPPH scavenging of all the coffee varieties increased (*p* < 0.05). With 200 μg/mL ascorbic acid, coffee bean extract from Jimma, Sidama, Yirgachefe, Nekemte, and Hararge scavenged the DPPH free radical by 98.76, 95.92, 99.92, 95.25, 96.94, 98.54%, respectively. For these extracts the respective IC50 values were 19.86, 20.22, 85.66, 55.43, 47.96, and 20.02 μg/mL. Overall, with all concentrations the Hararge coffee had comparable percentage inhibition of the DPPH free radical with ascorbic acid (*p* > 0.05). Additionally, the IC50 values of the Jimma and Hararge coffee varieties was comparable with ascorbic acid (*p* > 0.05). The DPPH scavenging capacity (73.33–84.16%) of Ethiopian coffee varieties was reported by (56) at 0.2 mg/mL extract concentration. Likewise, the antioxidant capacity of green coffee beans showed highly significant differences across districts of Southwest Ethiopia with percentage inhibition of DPPH between (67–73%) (15). Coffee has been noted as an effective *in vitro* and *ex vivo* antioxidant source and is the primary source of chlorogenic acid in the human diet (44). The entire antioxidant activity of coffee bean is influenced by its origin, harvesting, processing, and preparation (57). Growing origin significantly affected the coffee beans’ antioxidant activity, as shown in Table 3, which is in line with research by Khattak (58), Mishra et al. (59), and Dangles (60).

**Table 3:** DPPH free radical scavenging of Ethiopian green *Coffee arabica* varieties grown in different agro-ecological areas.

Concentration (μg/mL)	% inhibition					
	Ascorbic acid	Jimma Coffee	Sidama Coffee	Yirgachefe Coffee	Nekemte Coffee	Hararge Coffee
20	49.95 ± 0.03^fA^	48.59 ± 0.53^fA^	20.28 ± 0.38^fD^	37.34 ± 1.76^fC^	39.94 ± 1.14^fB^	49.02 ± 0.83^fA^
40	55.68 ± 0.53^eA^	55.81 ± 0.18^eA^	29.53 ± 0.18^eD^	45.98 ± 0.89^eC^	47.88 ± 1.89^eB^	55.84 ± 0.59^eA^
80	65.86 ± 0.16^dA^	66.75 ± 1.02^dA^	47.88 ± 0.56^dD^	56.93 ± 1.87^dC^	60.82 ± 1.33^dB^	67.70 ± 1.36^dA^
120	77.87 ± 0.09^cA^	75.60 ± 1.14^cB^	65.81 ± 0.07^cD^	72.87 ± 0.86^cC^	73.74 ± 0.66^cC^	75.72 ± 1.16^cB^
160	89.06 ± 0.25^bA^	87.76 ± 0.08^bB^	83.76 ± 1.04^bC^	84.34 ± 1.23^bC^	84.81 ± 0.18^bC^	86.75 ± 0.58^bB^
200	98.76 ± 0.14^aA^	95.92 ± 0.63^aB^	99.92 ± 0.64^aA^	95.25 ± 0.32^aB^	96.94 ± 0.59^aB^	98.54 ± 1.35^aA^
IC_50_ (μg/mL)	19.86 ± 0.05^D^	20.22 ± 1.44^D^	85.66 ± 0.63^A^	55.43 ± 1.09^B^	47.96 ± 1.07^C^	20.02 ± 1.06^D^

Data are expressed as mean ± standard deviation (*n* = 3) on dry basis. Mean values within a row and column with different block and small letter superscripts, respectively, indicate significant difference at *p* < 0.05 using one-way-analysis of variance (ANOVA), in mean comparisons using Duncan’s multiple range test. DPPH, 2,2-Diphenyl-1-picrylhydrazyl; IC50, 50% inhibitory concentration.

### Influence of Ethiopian traditional coffee roasting on biochemical composition

Due to its bioactive compound composition, the consumption of coffee can reduce colon and oral cancer, diabetes, liver disease, inhibits the oxidation of low density lipoprotein (LDL), cholesterol, protects against Parkinson’s disease and even reduces mortality risk (44). However, the profile and content of bioactive compounds depend mainly on roasting parameters, which range from 160 to 240°C and from 8 to 24 min. Ethiopian coffee roasting and brewing is unique and widely used in every household. However, there is dearth of reports on its influence on the biochemical composition of the local coffee varieties. As reported in Table 4, with all the coffee beans the roasting has reduced significantly the TPC (*p* < 0.05). The percentage reduction was; Jimma (24%), Sidama (26%), Nekemte (30%), Yirgacheffe (23%), and Hararge (29%). Similarly, Krol et al. (44) reported that the content of polyphenols was significantly affected by the intensity of the roasting process and the highest content was observed for fresh light-roasted beans, while the lowest for medium roasted. Sualeh et al. (15) reported a 15.33% significant reduction in overall antioxidant activity of green coffee bean by roasting at 200°C for 8 min. On the other hand, Mayer et al. (61) found that Colombian and Kenyan coffees with higher roasting degrees had higher concentrations of phenolic compounds, such as guaiacol.

**Table 4:** Influence of Ethiopian traditional coffee roasting on total phenols, chlorogenic acid, trigonelline, and caffeine content of Ethiopian *Coffee arabica* varieties grown in different agro-ecological areas.

Coffee origin	Coffee type	Total polyphenols (mg GAE/100 g)	Caffeine (g/100 g)	Chlorogenic acid (g/100 g)	Trigonelline (g/100 g)
Jimma	Green coffee	46.52 ± 0.82^*^	8.85 ± 0.00	45.95 ± 0.01^*^	12.88 ± 0.01^*^
	Roasted	35.56 ± 1.08	8.84 ± 0.00	7.84 ± 1.08	5.88 ± 1.00
Sidama	Green coffee	44.31 ± 4.68^*^	8.23 ± 0.00	45.29 ± 0.01^*^	13.56 ± 0.02^*^
	Roasted	32.87 ± 0.05	8.11 ± 0.01	9.38 ± 0.91	9.60 ± 0.66
Nekemte	Green coffee	44.55 ± 3.28^*^	10.18 ± 0.00	46.39 ± 0.01^*^	13.46 ± 0.01^*^
	Roasted	31.36 ± 0.19	9.88 ± 0.10	8.43 ± 0.69	7.33 ± 0.67
Yirgacheffe	Green coffee	34.25 ± 3.83^*^	10.38 ± 0.00	39.54 ± 0.01^*^	11.78 ± 0.00^*^
	Roasted	26.56 ± 0.91	9.92 ± 0.07	7.66 ± 0.84	7.27 ± 1.19
Hararge	Green coffee	39.02 ± 2.93^*^	7.55 ± 0.00	36.78 ± 0.02^*^	11.65 ± 0.00^*^
	Roasted	27.86 ± 1.01	7.45 ± 0.03	6.46 ± 0.89	7.64 ± 0.64

Data are expressed as mean ± SD (*n* = 3), on dry basis. ^*^indicates significant difference between green and roasted coffee beans using paired *t*-test (*p* < 0.05).

The highest significant percentage reduction upon roasting was in the chlorogenic acid content with all the coffee varieties (*p* < 0.05). The percentage reduction was: Jimma (83%), Sidama (79%), Nekemte (82%), Yirgacheffe (81%), and Hararge (82%). The average reduction was by 81% (Table 4). Likewise, Sualeh et al. (15) reported the maximum loss in biochemical content of coffee manifested in terms of percentage of chlorogenic acid by 54% due to roasting. Additionally, the trigonelline concentration varied significantly with all the coffee varieties during roasting (*p* < 0.05). The average reduction was 40%, with respective reductions of Jimma (54%), Sidama (29%), Nekemte (45%), Yirgacheffe (38%), and Hararge (34%) (Table 4). Sualeh et al. (15) reported a 7.69% reduction of trigonelline content in green coffee due to roasting. These reductions are due to the Maillard reaction some amount of chlorogenic acid and trigonelline changed to other form (62). However, the change in the caffeine content of coffee varieties after roasting was not statistically significant (Table 4). This is because the roasting did not change caffeine into volatile or to another chemical form. Similar observation confirmed that caffeine remained unchanged during roasting (63).

## Conclusion

Coffee quality and biochemical composition are believed to vary with geographical origin and method of processing. The results of the present study showed that coffee from five major producing areas of Ethiopia including Jimma/Limu, Sidama, Hararge, Nekemte, and Yirgacheffe possess considerable biochemical differences and antioxidant potential. The second hypothesis was as well confirmed, in which the roasting reduced the bioactive compounds in the coffee beans. Hararge coffee had the highest significant content of crude fat and crude protein. The total phenolic and flavonoid contents of the five coffee bean varieties differ significantly, with the highest contents in the Jimma and Nekemte beans. With all the tested concentrations, the Hararge coffee had comparable percentage inhibition of the DPPH free radical with ascorbic acid. The respective highest and lowest caffeine concentration was obtained in the Yirgachefe and Hararge coffee beans. The Jimma, Sidama, and Nekemte coffee varieties had the highest chlorogenic acid, while the Hararge variety had the lowest content. Likewise, the Jimma, Sidama, and Yirgachefe coffee varieties had the highest trigonelline contents. With all the coffee beans the roasting has reduced significantly the total TPC and other bioactive compounds. The highest significant percentage reduction upon roasting was in the chlorogenic acid content. However, the change in the caffeine content of coffee varieties after roasting was not statistically significant. Updated data across the years on the biochemical composition of coffee varieties can be used for evidence based climate change adaptation towards the production of quality coffee.

## Data availability statement

The original contributions presented in the study are included in the article/supplementary material, further inquiries can be directed to the corresponding author.

## Author contributions

DM: Conceptualization, Data curation, Formal analysis, Investigation, Methodology, Software, Validation, Visualization, Writing – original draft, Writing – review & editing. NR: Conceptualization, Project administration, Supervision, Visualization, Writing – review & editing. HW: Methodology, Software, Validation, Visualization, Writing – review & editing. PG: Conceptualization, Investigation, Methodology, Supervision, Validation, Writing – original draft, Writing – review & editing.
